# Early detection and management strategies for isolated splenic metastasis in cervical cancer: a case report

**DOI:** 10.3389/fonc.2025.1639109

**Published:** 2025-09-17

**Authors:** Na Zhang

**Affiliations:** The Department of Gynecologic Oncology, Chongqing University Cancer Hospital, Chongqing, China

**Keywords:** cervical cancer, spleen metastasis, splenectomy, chemotherapy, targeted therapy

## Abstract

A 55-year-old woman experienced 2 months of vaginal bleeding after intercourse. After gynecological examination, cervical biopsy, and imaging examinations including magnetic resonance imaging (MRI) and positron emission tomography/computed tomography (PET/CT), she was diagnosed with stage IIB cervical squamous cell carcinoma, which was associated with *human papillomavirus* (HPV) infection. The patient subsequently underwent robotic-assisted para-aortic lymphadenectomy, and the pathology results showed no evidence of cancer spread. She completed a treatment plan that included chemoradiotherapy, delivering a total radiation dose of 93.2 Gy along with concurrent cisplatin chemotherapy. Although a post-treatment evaluation indicated a partial remission (PR), follow-up imaging revealed unusual findings in the spleen, which were later confirmed to be metastatic cancer. The patient underwent a laparoscopic splenectomy, and the postoperative pathology confirmed the presence of squamous cell carcinoma metastasis. Genetic testing identified multiple somatic mutations and a high mutational burden in the tumor. After surgery, she received chemotherapy and targeted therapy. At present, her condition is stable, and she has survived for 12 months. This case highlights the complex nature of cervical cancer metastasis and the important role of genetic testing in developing personalized treatment plans.

## Introduction

Cervical cancer ranks as the fourth most frequently diagnosed cancer among women globally, especially in developing countries, where both the incidence and mortality rates are alarmingly high. The World Health Organization reports that cervical cancer accounts for over 300,000 deaths each year among women. The primary cause of cervical cancer is infection with human papillomavirus (HPV), particularly high-risk types such as HPV-16 and HPV-18, which play a significant role in its development (1). The clinical symptoms of cervical cancer can vary widely, with early signs often being subtle, including irregular vaginal bleeding, bleeding after intercourse, and unusual vaginal discharge. These symptoms are frequently overlooked, leading to delays in diagnosis (2). Despite recent advancements in cervical cancer screening and vaccination, the significance of early detection and prompt intervention remains crucial for improving patient survival rates (3).

This case report details the clinical journey of a 55-year-old postmenopausal woman who experienced vaginal bleeding after sexual intercourse and was eventually diagnosed with stage IIB cervical squamous cell carcinoma. This case is particularly noteworthy due to the occurrence of isolated splenic metastasis following standard treatment, a relatively rare event in cervical cancer cases. The report emphasizes the importance of a thorough differential diagnostic approach in clinical practice and highlights the need for careful monitoring and management of potential metastases during treatment. By analyzing this case, we aim to provide a reference for diagnosing and managing similar clinical situations in the future and to enhance the understanding of the metastatic pathways associated with cervical cancer.

## Case presentation

On June 20, 2022, a 55-year-old postmenopausal woman experienced vaginal bleeding after intercourse. The bleeding was light and appeared as bright red spotting. It persisted for 2 months. The patient reported no vaginal discharge, anal fullness, lower abdominal pain, or lumbosacral discomfort. There were no symptoms of dysuria, hematuria, urinary frequency, urgency, or constipation. No swelling or pain was observed in the lower limbs. Additionally, the patient had no chest pain, cough, or hemoptysis. The patient also reported no fatigue or weight loss. No family history of tumors was noted. During her initial gynecological examination, the external genitalia appeared normal, and the vagina was patent. However, the cervix showed nodularity, measuring approximately 2 cm in diameter. The left parametrium was noted to be shortened and thickened, while the right side exhibited good elasticity. The uterus was anteverted and of normal size, with no masses palpated in the adnexa. A cervical biopsy confirmed the presence of moderately differentiated HPV-related squamous cell carcinoma located at the 6 and 12 o’clock positions, along with a diagnosis of high-grade squamous intraepithelial lesion (HSIL; Cervical Intraepithelial Neoplasia (CIN) III) within the cervical canal. The immunohistochemical analysis showed P40(+), P53(+,10%), P16(+), Her-2(−), Ki-67(+,60%), and CK5/6(+). Based on the gynecological examination and cervical biopsy results, the preliminary diagnosis was cervical squamous cell carcinoma stage IIB (International Federation of Gynecology and Obstetrics (FIGO) 2018). The pathology was clear, and no differential diagnosis was needed. Subsequent pelvic enhanced magnetic resonance imaging (MRI) revealed a soft tissue mass in the cervix measuring approximately 4.1 × 3.4 × 5.0 cm, with unclear parametric spaces, suggesting that it involved the vaginal fornix and extended into the uterine body. Positron emission tomography/computed tomography (PET/CT) scan indicated cervical thickening with increased metabolic activity, recording a maximum standardized uptake value (SUV) of 12.6 and measuring approximately 3.5 × 3.0 × 4.4 cm, affecting the lower segment of the uterine body. Tumor biomarkers were elevated, with cancer antigen 125 (CA-125) at 22.80 U/mL (reference range, 0–16.0 U/mL), cancer antigen 19-9 (CA19-9) at 41.46 U/mL (reference range, 0–34.0 U/mL), squamous cell carcinoma antigen (SCC) at 4.60 ng/mL (reference range, 0–1.50 ng/mL), and cytokeratin 19 fragment at 5.92 ng/mL (reference range, 0–2.08 ng/mL).

On July 1, 2022, the patient underwent robotic-assisted surgery, which involved dissecting the para-aortic lymph nodes, releasing intestinal adhesions, and marking lymph nodes. Postoperative pathology confirmed that there was no cancer metastasis in the para-aortic lymph nodes, with all seven sampled nodes showing no signs of cancer.

From July 12 to August 25, 2022, the patient received fixed-field intensity-modulated radiotherapy, totaling 50.4 Gy delivered in 28 fractions of 1.8 Gy each. This treatment targeted the uterus, cervix, bilateral adnexa, bilateral parametrium, part of the vagina, and surrounding pelvic lymphatic drainage areas. Alongside this, the patient was administered five weekly doses of cisplatin on July 13, July 20, July 27, August 4, and August 28, 2022, with each dose being 40 mg/m². Additionally, the patient completed six sessions of painless three-dimensional brachytherapy on August 5, August 12, August 19, August 26, September 2, and September 7, 2022, with each session delivering 5.5 Gy using one tube with two spheres. As a result, the total cumulative dose from both external irradiation and brachytherapy reached 93.2 Gy. After treatment, no vaginal bleeding recurred in the patient. The primary side effects experienced during the chemoradiotherapy included nausea, vomiting, and grade III bone marrow suppression; however, these symptoms improved with symptomatic treatment.

A follow-up MRI conducted on September 20, 2022, revealed a mass-like soft tissue signal in the cervix, primarily located on the posterior wall, with dimensions of approximately 2.3 × 2.0 × 2.5 cm. The results from the cervical ThinPrep Cytology Test (TCT) showed no intraepithelial lesions or malignant cells, categorizing the findings as negative for intraepithelial lesions or malignancy (NILM). Additionally, testing for HPV type 16 returned positive, and the SCC level was noted to be elevated at 1.90 ng/mL. A cervical biopsy revealed inflammatory necrotic tissue alongside chronic inflammation of the mucosal lining. These findings led to an assessment of the therapeutic response as partial remission (PR). The patient is currently undergoing regular follow-up examinations, which have shown no signs of disease progression or recurrence.

**Figure 1:**
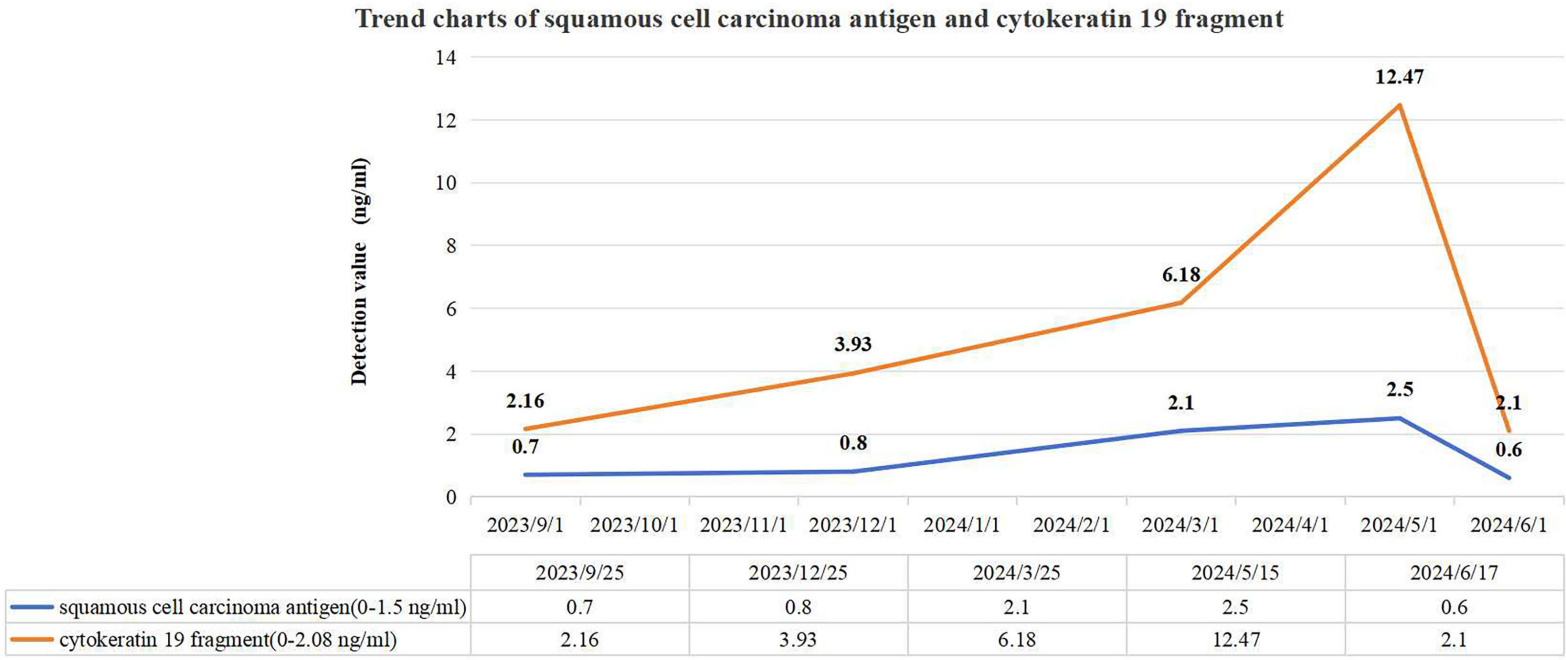
Trend charts of squamous cell carcinoma antigen and cytokeratin 19 fragment. Three months prior to the initial detection of a splenic lesion in December 2023, the level of cytokeratin 19 fragment was abnormally elevated, whereas the squamous cell carcinoma antigen level showed abnormal elevation 3 months after the appearance of the splenic lesion.

**Figure 2:**
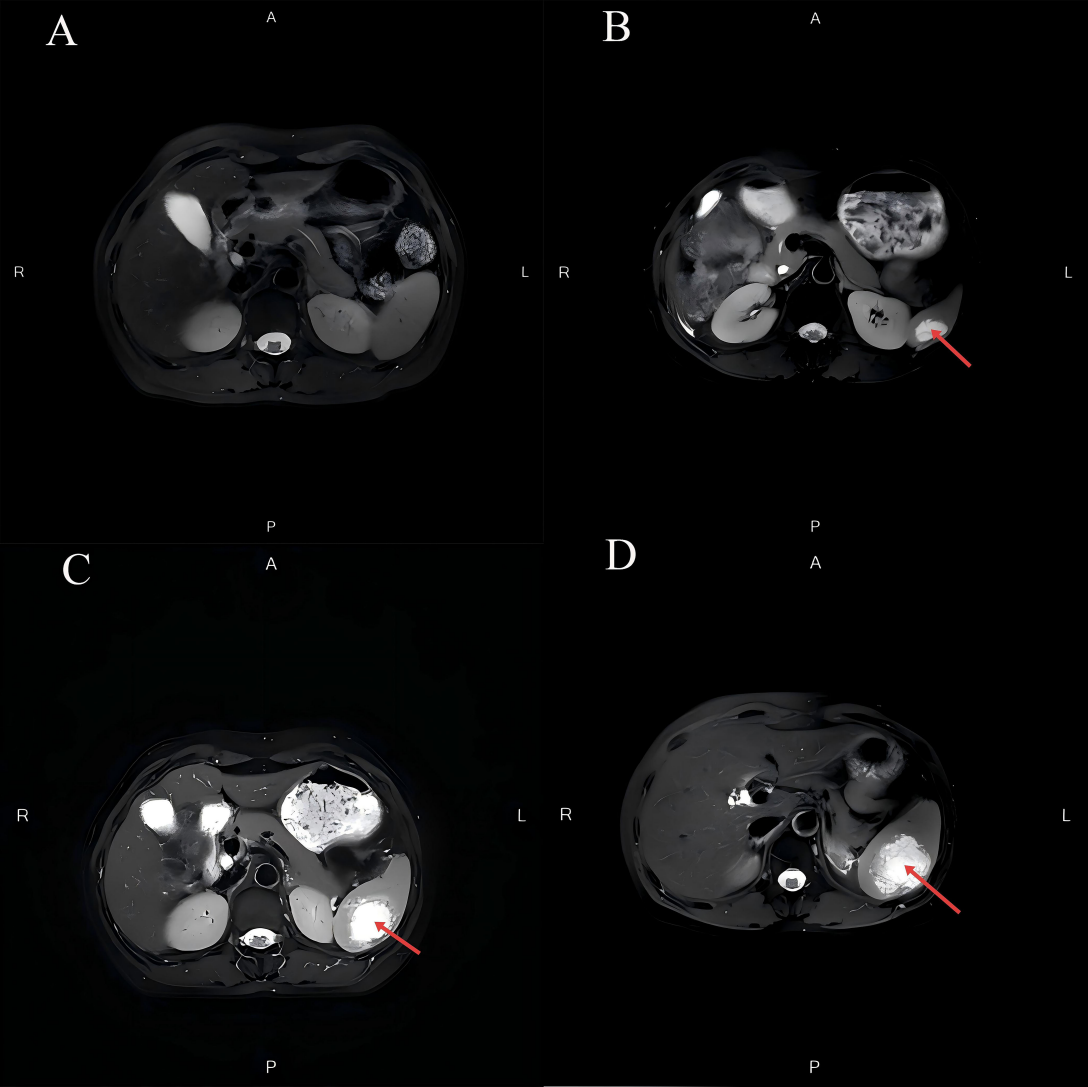
Changes in splenic lesions on MRI during the follow-up period. **(A)** The MRI in September 2023 revealed a normal-sized spleen with no abnormal signals or space-occupying lesions. **(B)** The MRI in December 2023 demonstrated an abnormal signal lesion measuring approximately 2.4 × 1.3 cm within the spleen. **(C)** The MRI in March 2024 revealed an abnormal signal lesion measuring approximately 4.0 × 3.0 cm within the spleen. **(D)** The MRI in May 2024 indicated that the spleen was not enlarged. A mass shadow measuring approximately 5.8 × 4.6 cm was observed within the splenic parenchyma, with ill-defined borders. After contrast enhancement, there was mild-to-moderate heterogeneous enhancement at the periphery of the mass, while the internal portion showed minimal enhancement.

**Figure 1** depicts the fluctuations in tumor markers throughout the follow-up period. The MRI in September 2023 revealed a normal-sized spleen with no abnormal signals or space-occupying lesions (**Figure 2A**). However, on December 26, 2023, an MRI identified a new isolated lesion in the spleen, measuring approximately 2.4 × 1.3 cm (**Figure 2B**). However, the patient exhibited no clinical symptoms. Further examinations were recommended to determine whether the lesions were primary or secondary, with the patient requesting continued monitoring. Subsequent follow-up assessments indicated a gradual increase in the size of the spleen lesion. An MRI performed on March 25, 2024, confirmed that the spleen lesion had grown to approximately 4.0 × 3.0 cm, indicating a notable increase from previous evaluations (**Figure 2C**).

**Figure 3:**
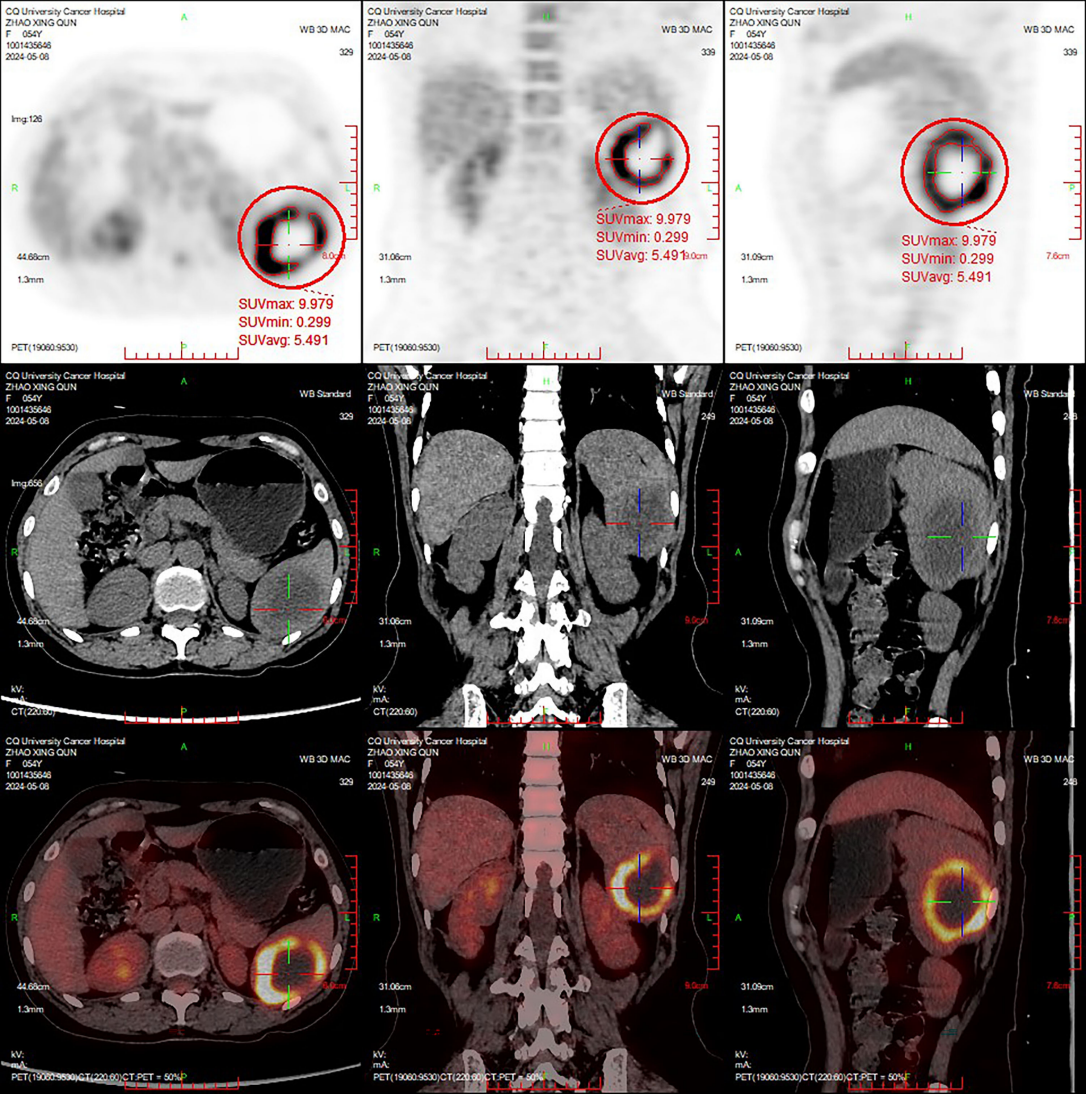
The PET/CT images acquired in May 2024. A patchy hypodense lesion was observed within the spleen, with ill-defined borders and a size measuring approximately 4.4 × 3.5 cm. The periphery of the lesion exhibits annularly elevated metabolic activity, with a maximum standardized uptake value (SUVmax) of approximately 10.0.

In May 2024, the patient reported intermittent dull pain in the left upper abdomen. The pain worsened during inhalation and persisted for 1 month. The pain was non-referred. Initially rated as 4 on the Numeric Rating Scale (NRS), the patient received oral lofentanil sustained-release tablets (2 tablets every 12 hours) for management. After treatment, the pain score had decreased to 1. Upon physical examination, the abdomen was found to be soft, with no tenderness, muscle rigidity, or rebound tenderness, and the spleen was not palpable. An MRI conducted on May 6, 2024, revealed that an isolated signal lesion in the spleen had increased in size to 4.6 × 4.0 cm, characterized by multiple septations and enhancement, which raised concerns about potential metastasis (**Figure 2D**). Following this, a PET/CT scan performed on May 8, 2024, identified a patchy low-density area in the spleen with indistinct borders, measuring approximately 4.4 × 3.5 cm, and an SUVmax of approximately 10.0 (**Figure 3**). On May 16, 2024, a CT scan showed that the spleen was not enlarged; however, it did reveal an isolated mass within the splenic parenchyma, measuring approximately 5.8 × 4.6 cm. This mass had indistinct borders and exhibited mild-to-moderate heterogeneous enhancement at its periphery, while the internal enhancement was minimal.

**Figure 4:**
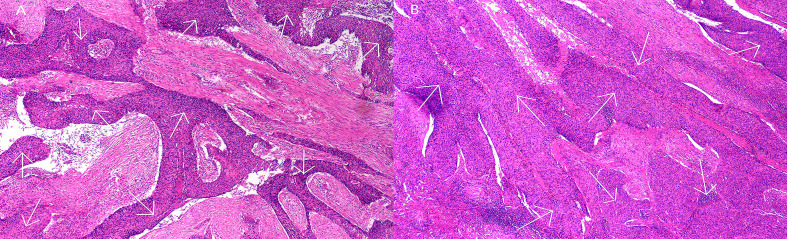
Pathological features following splenectomy. Hematoxylin and eosin (H&E) staining at ×200 magnification.The white arrows in the histological images indicate the deep purple areas representing squamous cell carcinoma. **(A)** Metastatic cancer lesions in splenic tissue; **(B)** Metastatic cancer lesions within splenic tumor tissue.

A multidisciplinary team of specialists recommended a splenectomy followed by postoperative anti-tumor therapies for the patient. On May 17, 2024, the patient underwent a laparoscopic total splenectomy. During the procedure, an enlarged spleen was observed, measuring 10.0 × 8.0 × 4.0 cm. The cut surface of the spleen revealed a grayish-white mass approximately 5.0 × 4.5 × 4.0 cm, which had a moderate consistency and exhibited necrotic areas. This mass was well-demarcated from the surrounding tissues and was located approximately 1.5 cm from the splenic hilum, close to the capsule. Post-surgical pathological analysis of both the spleen and tumor specimens confirmed the presence of metastatic cancer, with morphological and immunohistochemical characteristics consistent with squamous cell carcinoma metastasis, and clear margins were observed (**Figure 4**). The immunohistochemical profile showed P40(+), variable expression of P53, diffuse positivity for P16, CK5/6(+), and a Ki-67 proliferation index of 30%. The combined positive score (CPS) was estimated to be approximately 25. Genetic testing conducted on June 14, 2024, identified 24 somatic mutations, several of which were considered potentially clinically significant: the *NCOR1* gene intron 28 c.3812–1 G>T mutation (49.84% abundance), *PIK3CA* gene exon 10 p.E542K (c.1624G>A) missense mutation (39.69% abundance), *U2AF1* gene exon 2 p.S34F (c.101C>T) missense mutation (29.48% abundance), and *PLXNA1* gene intron 27 c.4871-1G>A mutation (26.15% abundance). The tumor mutational burden (TMB) was measured at 6.09 Muts/Mb, placing this case within the top 29% of cervical cancer instances. Additionally, microsatellite instability (MSI) testing indicated an MSI-L/MSS status.

**Figure 5:**
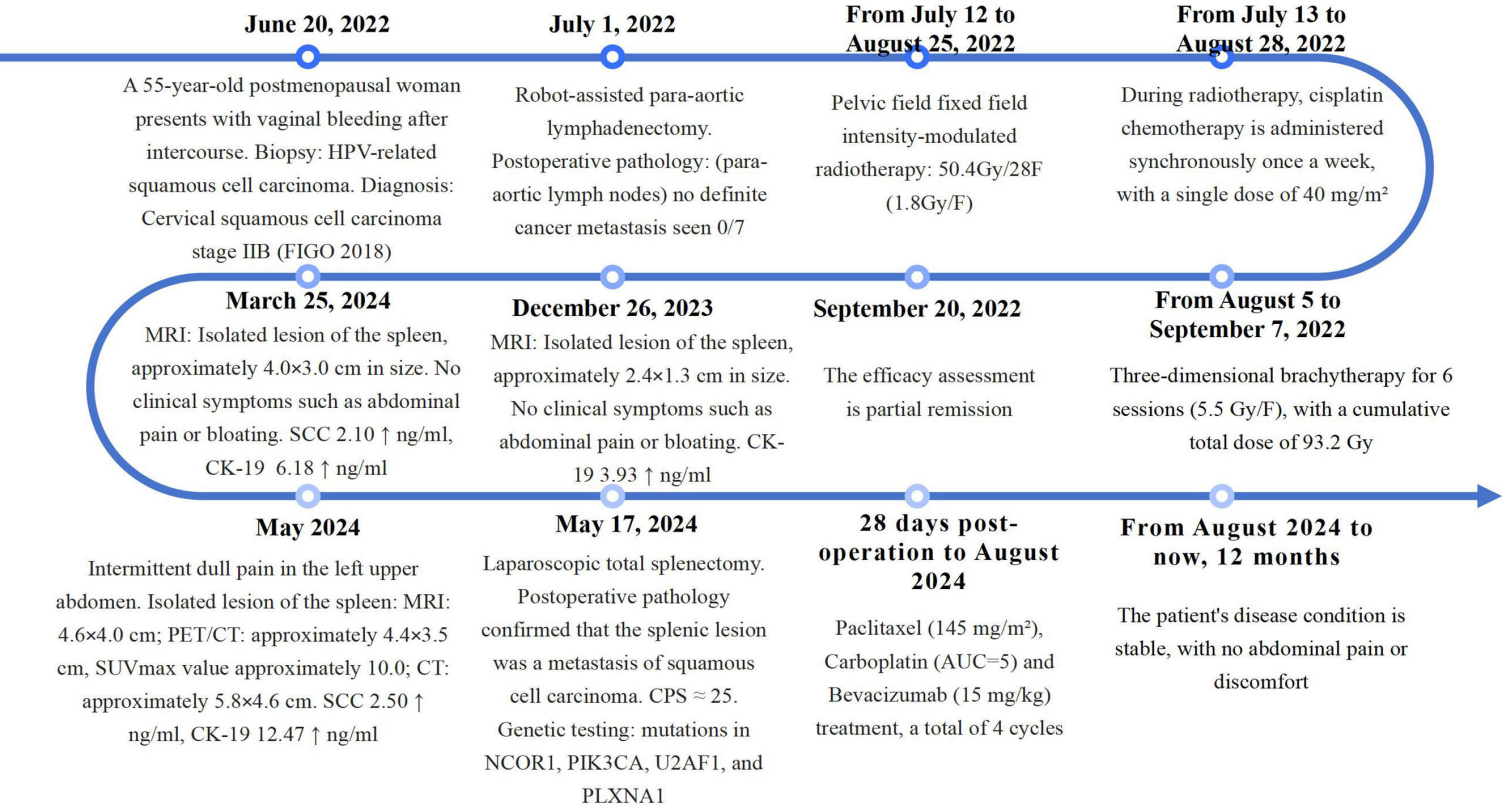
Timeline diagram of patient diagnosis and treatment. This figure illustrates the process of the patient from initial treatment to recurrence and post-recurrence treatment, including time, diagnosis, important examination and test results, treatment plan, and evaluation of treatment outcomes.

The patient refused immunotherapy. The patient subsequently received a treatment regimen consisting of paclitaxel (145 mg/m^2^), carboplatin (Area Under the Curve (AUC) = 5), and bevacizumab (15 mg/kg) over four cycles. The last chemotherapy session occurred in August 2024. After completing the treatment, the patient had no abdominal pain for 12 months (**Figure 5**). The main side effects during the patient’s treatment were nausea, vomiting, myelosuppression (grade II-III), fatigue, mild hypertension (142/91 mmHg), and mild numbness in the hands and feet, which improved with symptomatic treatment.

## Discussion

Interestingly, the spleen is an unusual site for metastasis from cervical cancer, with only a few cases reported in the literature (4). One significant factor in this process is genomic instability, which arises from HPV infection and is believed to play a crucial role in the spread of cervical cancer. Although there have been improvements in early detection and treatment options that allow many patients to receive timely care, the challenge of tumor metastasis remains a significant hurdle for achieving better prognoses. In the case discussed, the patient initially responded well to standard therapies; however, the later development of isolated splenic metastasis underscored ongoing challenges in managing the disease.

Research has shown that cervical cancer typically spreads through lymphatic and blood routes. The lymphatic spread mainly affects the pelvic and para-aortic lymph nodes, while hematogenous metastasis usually occurs in the later stages of the disease. Studies have also revealed that cervical cancer cells can enter the bloodstream, resulting in metastasis to various organs, including the lungs, liver, and bones (5).

Primary splenic tumors are extremely rare, and isolated splenic metastases from solid tumors are found in approximately 1% of autopsy cases (6). Typically, these splenic metastases originate from cancers such as breast, lung, and melanoma. While primary splenic tumors are uncommon and metastases generally come from other cancers, the specific mechanisms that lead to splenic metastasis in cervical cancer are not well understood.

Recent studies have indicated that the spleen, a key part of the immune system, may have a complex role in tumor metastasis. Several factors could contribute to this process: first, the unique structure of splenic blood sinuses can trap tumor cells, allowing cervical cancer cells in the bloodstream to enhance their retention and growth in the spleen through interactions with vascular endothelial cells (5, 7). Second, the spleen is rich in immune cells, which means that tumor cells must evade immune responses to survive. The microenvironment within the spleen may provide vital nutrients and signaling pathways that support tumor cell growth, thereby increasing their survival and proliferation. Third, specific genetic mutations may aid in the migration or adaptation of tumor cells within the spleen. Lastly, the peritoneum serves as an important pathway for the metastasis of cervical cancer cells into the abdominal cavity. Research has suggested that cervical cancer cells can affect the metastatic behavior of organs like the spleen through peritoneal dissemination, particularly as the cancer progresses and peritoneal infiltration facilitates the spread of tumor cells (8).

The occurrence of splenic metastasis in patients with cervical cancer is relatively rare, with the first documented case reported by Brufman et al. in 1977 (9). A review of existing cases reveals that the median age of patients with splenic metastasis from cervical cancer is 47 years, with a standard deviation of 10.32 years. Among these patients, 7.69% are classified as stage IB1, 7.69% as stage IIA, 50.00% as stage IIB, 7.69% as stage IIIB, 3.85% as stage IIIC, and 7.69% as stage IVA, indicating that stage IIB is the most commonly associated stage of cervical cancer linked to secondary splenic metastasis. In terms of histological classification, squamous cell carcinoma accounts for 69.23% of cases, adenocarcinoma for 23.08%, and adenosquamous carcinoma for 7.69% (**Table 1**) (4, 6, 8–30). The presence of splenic metastasis generally indicates disease progression and can present various clinical manifestations that may impact treatment strategies. Complications associated with splenic metastasis include splenomegaly with pain, thrombosis of the splenic vein, and the risk of splenic rupture (1, 20).

**Table 1:** A literature review on cases of cervical cancer with splenic metastasis.

Author	Year of publication	Age	Stage	Pathology	Initial treatment	PFS (month)	Symptoms	Metastasis	Splenic metastatic lesion size	Image	Surgery after metastasis	Chemotherapy after metastasis	Survival (months)
Aitelhaj M et al. (10)	2014	55	IIB	Squamous cell carcinoma Poorly differentiated	Chemoradiotherapy	8	A painful nodule in her left breast and left upper quadrant abdominal pain	Isolated splenic metastasis and left breast metastasis	10 cm at the largest diameter	CT	NA	Paclitaxel and cisplatin	3 months, died
AlQattan AS et al. (8)	2021	54	IIIB	Adenocarcinoma Moderately differentiated	Chemoradiotherapy	36	Asymptomatic	Isolated splenic metastasis	3.5 cm × 3.2 cm	MRI PET/CT	Distal pancreatectomy and splenectomy	Paclitaxel and carboplatin	More than 36 months, alive
Bacalbasa N et al. (11)	2017	31	IIB	Squamous cell carcinoma Moderately differentiated	Neoadjuvant chemotherapy and radiation therapy followed by hysterectomy	18	Asymptomatic	Isolated splenic metastasis	5 cm × 5 cm	NA	Splenectomy	Paclitaxel and cisplatin	12 months, alive
Brufman G et al. (9)	1977	43	NA	Squamous cell carcinoma	NA	60	NA	Isolated splenic metastasis	NA	NA	NA	NA	NA
Bhardwaj S et al. (12)	2008	50	IIB	Squamous cell carcinoma	Radiotherapy	41	Pain and heaviness in the left hypochondrium	Isolated splenic metastasis	7.5 cm × 5.5 cm × 1 cm	USG	Splenectomy	Chemotherapy	48 months, alive
Campagnutta et al. (13)	1992	47	IIB	Adenocarcinoma	Radical hysterectomy	60	Left-sided abdominal pain	Isolated splenic metastasis	16 cm × 9 cm × 6.5 cm	CT Hepatosplenic scintigraphy	Splenectomy	NA	7 months, alive
Carvalho L et al. (14)	1997	47	IIB	Squamous cell carcinoma Moderately differentiated	Radiotherapy	50	Persistent left hypochondrium pain radiating to the ipsilateral shoulder and weight loss	Isolated splenic metastasis	19 cm	CT	Exploratory laparotomy	Vincristine, methotrexate, bleomycin, and cisplatin	15 months, alive
Céline Petit et al. (15)	2003	41	IIB	Adenocarcinoma Poorly differentiated	Radiotherapy, chemotherapy, and surgery	36	Asymptomatic	Isolated splenic metastasis and presacral level metastasis	6.9 cm × 4.6 cm	MRI	Laparoscopic splenectomy	Paclitaxel and carboplatin	14 months, died
Di Donato V et al. (16)	2010	30	IVA	Squamous cell carcinoma Moderately differentiated	Neoadjuvant chemotherapy and radical hysterectomy with bilateral adnexectomy	30	Abdominal pain mainly localized in the left upper quadrant	A mass located in the proximity of the splenic hilum involving the gastric wall, pancreas, and splenocolic ligament	2 cm	PET/CT	Splenectomy, distal pancreatectomy, and partial gastric resection	Platinum-based chemotherapy	12 months, alive
Dixit J et al. (17)	2016	46	IB1	Squamous cell carcinoma Moderately differentiated	Radical hysterectomy and chemoradiotherapy	17	Fever and anorexia	Isolated splenic metastasis and pelvic mesentery metastasis	8 cm × 6 cm × 4.5 cm	PET/CT	Underwent resection of a segment of ileum with the mesenteric lesion and omentum and bladder peritoneum laparoscopically and splenectomy	Paclitaxel and cisplatin	Alive
Filipescu A et al. (18)	2018	30	IIB	Squamous cell carcinoma	Neoadjuvant radiochemotherapy followed by total radical hysterectomy	8	NA	Isolated splenic metastasis	NA	Not documented	Splenectomy	NA	8 months, alive
Goktolga U et al. (19)	2004	45	IIA	Squamous cell carcinoma Poorly differentiated	Radical hysterectomy and adjuvant radiotherapy	36	Abdominal fullness and pain in the left hypochondriac region	Isolated splenic metastasis and metastasis to hepatic artery and vein, stomach, left kidney, and adrenal gland	8 cm	USG CT	Explorative laparotomy was performed, but splenectomy or debulking surgery could not be done	Cisplatin and 5-FU	12 months after the second surgery, died
GUPTA T et al. (20)	2006	41	IIB	Adenosquamous Poorly differentiated	Chemoradiotherapy	10	Vague dyspeptic and pulmonary symptoms, moderate pallor, and hepatomegaly	Multiple hepatosplenic lesions and metastasis to the liver, celiac region, para-aortic lymph nodes, multiple thoracic vertebrae, and right humerus	Multiple lesions	USG CT PET/CT	NA	Paclitaxel and carboplatin	NA
Kim JH et al. (21)	2008	46	IIB	Adenocarcinoma	Chemoradiotherapy	11	Asymptomatic	Isolated splenic metastasis	2.4 cm	MRI	Splenectomy	Paclitaxel and carboplatin	18 months, alive
Kim JH et al. (21)	2008	54	IIB	Squamous cell carcinoma	Chemoradiotherapy	10	Asymptomatic	Isolated splenic metastasis	3 cm	CT PET/CT	Splenectomy	Paclitaxel and carboplatin	22 months, alive
Klamminger GG et al. (22)	2023	37	IIA	Adenosquamous carcinoma Poorly differentiated	Radical hysterectomy with chemoradiotherapy	17	Intermittent left-sided abdominal pain	Isolated, capsulated lesion	NA	CT MRI PET/CT	Splenectomy	Carboplatin, paclitaxel, pembrolizumab, and bevacizumab, followed by maintenance pembrolizumab and bevacizumab	10 months, alive
Klein B et al. (6)	1987	28	IIB	Squamous cell carcinoma	Radiotherapy	54	Pain in her left upper abdomen	Isolated splenic metastasis adhering to the kidney, left diaphragm, pancreas, and left adrenal gland	NA	CT	Splenectomy, left nephrectomy, distal pancreatectomy, and excision of the diaphragm	Intraperitoneal chemotherapy	Alive
Kumar A et al. (4)	2022	46	NA	Squamous cell carcinoma	Hysterectomy and chemoradiotherapy	7	Small bowel obstruction	Pelvic mass and peritoneal deposits	Cystic lesions	CT PET/CT	Exploratory laparotomy was undertaken with a diversion loop ileostomy	Palliative chemotherapy	NA
Liu Q et al. (23)	2020	57	IIB	Squamous cell carcinoma Moderately to poorly differentiated	Chemoradiotherapy	60	Left upper abdominal distension and anorexia	Isolated splenic metastasis	5.0 cm × 6.5 cm	CT	Splenectomy	Paclitaxel and cisplatin	46 months, died
Pang LC (24)	2004	50	IIA	Squamous cell carcinoma Moderately differentiated	Radical hysterectomy followed by radiotherapy	60	Asymptomatic	Isolated splenic metastasis	5.5 cm × 3.7 cm × 3.5 cm	CT MRI PET/CT	Laparoscopic splenectomy	Cisplatin	5 months, alive
Sharma P et al. (25)	2014	52	IIIB	Squamous cell carcinoma	Radical hysterectomy followed by radiotherapy	36	Pain and heaviness in left hypochondrium	Massive solid cystic splenomegaly and metastatic involvement of the pancreas and left supraclavicular lymph nodes	Massive solid cystic splenomegaly	PET/CT	NA	Paclitaxel and carboplatin	4 months, died
Shankar ST et al. (26)	2020	55	IVA	Squamous cell carcinoma Moderately differentiated	Chemoradiotherapy	36	Asymptomatic	Isolated splenic metastasis	2.1 cm × 3.7 cm	PET/CT	Splenectomy	NA	12 months, alive
Taga S et al. (27)	2014	49	IIB	Undifferentiated carcinoma	Chemoradiotherapy	10	Asymptomatic	Isolated splenic metastasis	NA	CT	Splenectomy	Nedaplatin	20 months, alive
Theocharopoulos C et al. (28)	2025	47	IIIC	Squamous cell carcinoma	Chemoradiotherapy	8	Weakness, tiredness, and shortness of breath, severely anemic	Isolated splenic metastasis appeared to directly invade the fundus of the stomach	6.8 cm × 6.8 cm	CT	Splenectomy, distal pancreatectomy, longitudinal gastrectomy, and pyloroplasty	Cisplatin, paclitaxel, and pembrolizumab	8 months, died
Valls et al. (29)	1992	55	NA	Adenocarcinoma	Radical hysterectomy and radiotherapy	14	NA	NA	NA	CT	Splenectomy	NA	NA
Villalón-López JS et al. (30)	2014	76	IB1	Adenocarcinoma Moderately differentiated	Hysterectomy and radiotherapy	24	Abdominal pain	Two solid lesions	5.3 cm × 5.5 cm and another greater than 2.1 cm × 2.2 cm	CT	Splenectomy	Paclitaxel and cisplatin	12 months, alive

PFS, progression-free survival; CT, computed tomography; NA, not available; MRI, magnetic resonance imaging; PET/CT, positron emission tomography/computed tomography; USG, ultrasound sonography; 5-FU, fluorouracil.

According to literature reports, the median survival time for patients with splenic oligometastasis from cervical cancer ranges from 5 to 30 months (15.86 ± 7.95 months) (23). In the case presented, the patient experienced a progression-free survival interval of 15 months from the end of cervical cancer treatment to the development of isolated splenic metastasis. Symptoms related to splenic metastasis in cervical cancer often present subtly, with patients frequently experiencing non-specific signs such as splenomegaly, which may result in abdominal pain or discomfort (4, 8). In some cases, systemic symptoms like fatigue, anemia, decreased appetite, and weight loss can also occur (4). These symptoms can mask other underlying health issues, making accurate diagnosis more challenging (31). For instance, 15 months after treatment, imaging studies revealed isolated splenic lesions in a patient; however, these lesions did not correlate with any clinical symptoms or signs at that time. It was not until 20 months post-treatment that the patient began to report discomfort and dull pain in the left upper quadrant of the abdomen, coinciding with imaging that showed a significant increase in both the size and extent of the splenic lesions. Clinical symptoms appeared nearly 5 months after imaging abnormalities were first detected, underscoring the vital role of routine imaging evaluations.

Common imaging techniques used in this context include ultrasound, CT, and MRI (5). CT scans often reveal splenomegaly and irregular margins, with findings that may indicate low-density areas or masses, which are crucial for assessing the size and location of tumors (32). MRI excels in imaging soft tissues, making it particularly useful for evaluating the spleen and surrounding structures (4, 5, 8). Additionally, PET/CT assesses the metabolic activity of the spleen, which helps in identifying metastatic lesions (4). PET/CT is recognized for its increased sensitivity in detecting these uncommon metastatic sites (4, 8). In this case, the patient underwent a comprehensive series of imaging evaluations, including CT, MRI, and PET/CT, which revealed isolated splenic lesions and significantly improved the accuracy of the diagnosis regarding splenic metastasis.

Individuals with splenic metastasis from cervical cancer often show non-specific changes in blood tests, such as anemia, low white blood cell counts, and reduced platelet counts, which suggest impaired splenic function (5, 33). However, in this case, the patient’s laboratory results did not indicate these common abnormalities. Some patients may also have elevated tumor markers like SCC, carcinoembryonic antigen (CEA), CA-125, CA19-9, and cytokeratin 19 fragment (4, 33). Although these markers are not specific to splenic metastasis, they can be useful in assessing disease progression and response to treatment (2, 4, 34). Notably, elevated tumor markers, particularly SCC, can predict metastasis with an accuracy of 46% to 92%, often being detectable up to 6 months before clinical signs appear, typically emerging 2 to 7.8 months earlier (35). In this case, imaging evaluation revealed a mild increase in cytokeratin 19 fragment 3 months before the splenic lesions were discovered. Meanwhile, SCC levels rose 3 months after the detection of spleen abnormalities. Both SCC and cytokeratin 19 fragment showed progressive elevation during serial tumor marker monitoring. Regular imaging assessments demonstrated gradual enlargement of the splenic lesion. Monitoring tumor markers dynamically can aid in condition evaluation.

The most reliable method to diagnose spleen metastasis is organ biopsy. In cervical cancer, spleen metastasis is closely associated with the aggressive behavior of tumor cells. In recent years, genetic testing and programmed death-ligand 1 (PD-L1) analysis have garnered significant attention in metastatic cervical cancer research. Studies have indicated that patients with spleen metastasis exhibit significantly elevated PD-L1 expression levels. This elevation may result from tumor cells suppressing T-cell activity to evade immune surveillance (4). In this case, the patient showed high PD-L1 expression with a CPS of approximately 25, making additional monitoring unnecessary. However, there is currently insufficient evidence to support routine dynamic monitoring of PD-L1. Re-evaluation of PD-L1 status may provide justification for adjusting treatment regimens only when disease progression or immune therapy resistance occurs. Immune checkpoint inhibitors target the programmed cell death protein 1 (PD-1)/PD-L1 pathway and show promising therapeutic potential for cervical cancer treatment. They demonstrate remarkable efficacy, especially in PD-L1-positive patients.

Genetic evaluations have identified four clinically significant mutations: *NCOR1*, *PIK3CA*, *U2AF1*, and *PLXNA1*. The expression of *NCOR1* may influence how cervical cancer cells metastasize to the spleen. Previous studies have shown that *NCOR1* can inhibit the transcription of p53, which in turn facilitates the progression and spread of cervical cancer (36). Additionally, mutations in *PIK3CA* are known to increase the invasiveness of tumor cells, promoting their migration to the spleen (34, 37). These mutations can lead to changes in the cytoskeleton and intercellular adhesion properties, enhancing the motility of cancer cells and allowing them to spread to the spleen through the bloodstream or lymphatic system (38). Therefore, *PIK3CA* mutations not only serve as indicators of cervical cancer onset but also may suggest a heightened risk for splenic metastasis. Similarly, mutations in *U2AF1* are significantly linked to the increased invasiveness and metastatic potential of tumor cells (39). These mutations may affect the development of various tumors by altering the splicing of important oncogenes, indicating their potential as biomarkers for assessing the risk of splenic metastasis in patients with cervical cancer (40). Furthermore, *PLXNA1* is involved in regulating critical processes such as the tumor microenvironment, angiogenesis, and epithelial–mesenchymal transition through various molecular pathways, thereby promoting tumor growth, migration, and metastasis (41).

Cervical cancer management requires a collaborative approach that brings together a multidisciplinary team, particularly in cases where splenic metastasis is present. This multidisciplinary treatment (MDT) framework leverages the expertise of professionals from various fields, such as gynecological oncology, radiation oncology, medical oncology, imaging, and pathology, to create tailored therapeutic strategies for each patient. Research has indicated that this coordinated effort significantly improves treatment effectiveness and increases survival rates for those affected by the disease (42).

Oligometastatic cervical cancer is defined by the presence of a limited number of metastatic lesions, typically no more than five. Importantly, there should be no local–regional recurrence, meaning that the tumor does not involve the uterus, cervix, upper third of the vagina, or pelvic wall (43). Treatment modalities, including surgical intervention, radiotherapy, and chemotherapy, should be customized based on the patients’ disease stage, the severity of splenic metastasis, and their overall health condition. Studies have shown that patients in the oligometastatic recurrence group have better progression-free survival (PFS) and disease-specific survival (DSS) than those in the metastatic recurrence group, regardless of the treatment regimen used. Within the oligometastatic recurrence cohort, surgical resection is linked to a lower risk of secondary cancer and significantly longer PFS (44).

Although splenic metastasis from cervical cancer is rare, surgical resection may offer a potentially curative option if the patient’s health is stable and the tumor is limited to the spleen, as supported by a documented case (44). The literature suggests that surgical removal of isolated splenic metastasis is typically performed using either laparoscopic or open surgical techniques. Postoperative pathological evaluations can confirm the nature and extent of the metastasis, which is crucial for guiding further treatment decisions (4, 8). In this case, the patient was diagnosed with metastatic squamous cell carcinoma following a splenectomy. Consequently, early identification of oligometastatic status and timely intervention are crucial to improving patient prognosis. However, these outcomes are closely linked to the patients’ physical condition, the location of metastases, and their response to treatment.

In recent years, the exploration of tumor biomarkers has greatly advanced the field of targeted therapies. Targeted agents, such as anti-*VEGF* medications like bevacizumab, are used alongside chemotherapy to improve patient outcomes by blocking the blood supply to tumors and slowing their growth, particularly in local tumor management and in delaying disease progression (45–47). In this case, the patient underwent a splenectomy followed by a treatment regimen that included paclitaxel, carboplatin, and bevacizumab. As of the submission date, the patient is alive and has shown no signs of recurrence or disease progression for nearly 9 months. Genetic testing has revealed several potentially significant mutations that could guide future treatment strategies. Research has suggested a link between TMB and the success of immunotherapy, which may lead to more personalized treatment approaches (48). Future studies should continue to investigate how genetic mutations impact treatment decisions and explore new targeted and immunotherapeutic options to improve the prognosis for patients with cervical cancer and splenic metastasis.

## Conclusion

This case highlights the complex pathological mechanisms and treatment responses seen in patients with cervical squamous cell carcinoma, underscoring the vital role of early detection and monitoring of tumor metastasis in clinical practice. Recent advancements in medical technologies, especially in imaging and biomarker identification, have opened new avenues for the early detection and evaluation of splenic metastasis in cervical cancer. Future research should focus on several important areas: first, clarifying the mechanisms behind splenic metastasis and exploring how tumor cells survive and proliferate within the spleen. Second, a deeper analysis is needed to understand how the splenic microenvironment influences the behavior of cervical cancer cells, particularly in terms of immune evasion and their ability to metastasize. Furthermore, clinical trials should aim to enhance follow-up care for patients with splenic metastasis by evaluating the effectiveness of different treatment regimens in prolonging survival. These efforts will equip clinicians with more practical treatment guidelines for managing cervical cancer patients facing splenic metastasis. Therefore, the study of splenic metastasis remains a crucial area for ongoing investigation.

In summary, the exploration of splenic metastasis in cervical cancer offers important theoretical insights and practical implications for clinical practice. This research encourages the merging of clinical applications with foundational studies through collaborative efforts across various disciplines. Future research should focus on identifying prognostic indicators and biomarkers linked to splenic metastasis in cervical cancer, with the goal of developing more accurate predictive tools for clinical application.

## Data Availability

The original contributions presented in the study are included in the article/supplementary material. Further inquiries can be directed to the corresponding author.

## References

[1] Makhdoom H . Merkel cell polyomavirus and their association with the pathogenesis of cervical squamous cell carcinomas and adenocarcinomas: A review article. Ethiop J Health Sci. (2023) 33:711–20. doi: 10.4314/ejhs.v33i4.18, PMID: 38784202 PMC11111187

[2] Wang Y Yuan S Ma J Liu H Huang L Zhang F . Substance P is overexpressed in cervical squamous cell carcinoma and promoted proliferation and invasion of cervical cancer cells. Eur J Histochem. (2023) 67:3746. doi: 10.4081/ejh.2023.3746, PMID: 37522867 PMC10476533

[3] Tariq B Phillips S Biswakarma R Talaulikar V Harper JC . Women’s knowledge and attitudes to the menopause: a comparison of women over 40 who were in the perimenopause, post menopause and those not in the peri or post menopause. BMC Womens Health. (2023) 23:460. doi: 10.1186/s12905-023-02424-x, PMID: 37648988 PMC10469514

[4] Kumar A Upadhyay A Pandey V Kumar B . Mitra S. A rare case of splenic metastasis from squamous cell carcinoma of the cervix detected on 18F-fluorodeoxyglucose PET/CT. Cureus. (2022) 14:e31974. doi: 10.7759/cureus.31974, PMID: 36589186 PMC9795960

[5] Fujisawa K Maeda H Aida M Kawanishi Y Munekage M Kitagawa H . Two cases of splenic tumors safely resected by hand-assisted laparoscopic splenectomy. Gan To Kagaku Ryoho. (2024) 51:1684–6., PMID: 39948966

[6] Klein B Stein M Kuten A Steiner M Barshalom D Robinson E . Splenomegaly and solitary spleen metastasis in solid tumors. Cancer. (1987) 60:100–2. doi: 10.1002/1097-0142(19870701)60:1<100::aid-cncr2820600118>3.0.co;2-9, PMID: 3581023

[7] Lam KY Tang V . Metastatic tumors to the spleen: a 25-year clinicopathologic study. Arch Pathol Lab Med. (2000) 124:526–30. doi: 10.5858/2000-124-0526-MTTTS, PMID: 10747308

[8] AlQattan AS Alqutub AA Masoudi JH Alassaf MAM Mansi N . Splenic oligometastasis from cervical adenocarcinoma three years after disease free survival: A case report and a review of literature. Ann Med Surg (Lond). (2021) 72:103144. doi: 10.1016/j.amsu.2021.103144, PMID: 34934488 PMC8654797

[9] Brufman G Biran S Goldschmidt Z Freund U . Solitary metastatic involvement of the spleen in squamous cell carcinoma of the cervix. Harefuah. (1977) 92:349–50., PMID: 863327

[10] Aitelhaj M Khoyaali SL Boukir A Elkabous M Abahssain H Mrabti H . Breast and splenic metastases of squamous cell carcinoma from the uterine cervix: a case report. J Med Case Rep. (2014) 8:359. doi: 10.1186/1752-1947-8-359, PMID: 25366471 PMC4234522

[11] Bacalbasa N Balescu I Marcu M Oprescu DN Anca AF . Solitary splenic metastasis after surgically-treated cervical cancer -A case report and literature review. Anticancer Res. (2017) 37:2615–8. doi: 10.21873/anticanres.11607, PMID: 28476835

[12] Bhardwaj S Mahajan D Vir Gupta Y . Metastatic squamous cell carcinoma of the cervix presenting as a splenic cyst. J K Sci. (2008) 10:146–8.

[13] Campagnutta E Zarrelli A Stefanutti V Cimitan M Querin F Scarabelli C . Metastasi splenica isolata in un caso di adenocarcinoma del collo uterino. Caso clinico [Isolated splenic metastasis in a case of adenocarcinoma of the uterine cervix. A clinical case. Minerva Ginecol. (1992) 44:667–70. 1491776

[14] Carvalho L Azevedo I Salgado L Ferreira ES Henrique R de Carvalho RG . Squamous cell carcinoma of the cervix metastatic to the spleen–case report. Gynecol Oncol. (1997) 67:107–10. doi: 10.1006/gyno.1997.4814, PMID: 9345365

[15] Petit C Demolin G Stamatiou A Saadi S . Vandingenen T.Recurrence of poorly differenciated cervical cancer by single splenic metastasis: case report and literature review. Case Rep Clin Med. (2023) 12:93–101. doi: 10.4236/crcm.2023.124013

[16] Di Donato V Palaia I Perniola G Polidori N Burratti M Besharat A . Splenic metastasis from cervical cancer: case report and review of the literature. J Obstet Gynaecol Res. (2010) 36:887–90. doi: 10.1111/j.1447-0756.2010.01210.x, PMID: 20690225

[17] Dixit J Mohammed N Shetty P . Splenic metastasis from cancer of uterine cervix-a rare case. Indian J Surg Oncol. (2016) 7:479–83. doi: 10.1007/s13193-016-0564-7, PMID: 27872541 PMC5097777

[18] Filipescu A Balescu I Bacalbasa N . Upper abdominal resection for isolated metastatic lesions in recurrent cervical cancer. Anticancer Res. (2018) 38:1659–63. doi: 10.21873/anticanres.12398, PMID: 29491099

[19] Goktolga U Dede M Deveci G Yenen MC Deveci MS Dilek S . Solitary splenic metastasis of squamous cell carcinoma of the uterine cervix: a case report and review of the literature. Eur J Gynaecol Oncol. (2004) 25:742–4., PMID: 15597857

[20] Gupta T Nair N Fuke P Bedre G Basu S Shrivastava SK . Splenic metastases from cervical carcinoma: a case report. Int J Gynecol Cancer. (2006) 16:911–4. doi: 10.1111/j.1525-1438.2006.00220.x, PMID: 16681784

[21] Kim JH Choi YD Lee JH Nam JH Juhng SW Koh YS . Solitary splenic metastases from uterine cervical cancer: case reports and review of the literature. Korean J Pathol. (2008) 42:317–22.

[22] Klamminger GG Burgard C Rosar F Altmeyer K Malinowski M Nigdelis MP . Unusual case of splenic metastasis in adenosquamous carcinoma of the cervix uteri: diagnosis and treatment considerations. Am J Case Rep. (2023) 24:e941600. doi: 10.12659/AJCR.941600, PMID: 38062677 PMC10720923

[23] Liu Q Wang M Gayam V Li XL Wang FC Pan CQ . The clinical course and management of cervical cancer with splenic metastasis: Case report and review of the literature. Clin Case Rep. (2020) 9:689–93. doi: 10.1002/ccr3.3621, PMID: 33598227 PMC7869355

[24] Pang LC . Solitary recurrent metastasis of squamous cell carcinoma of the uterine cervix in the spleen: case report. South Med J. (2004) 97:301–4. doi: 10.1097/01.SMJ.0000078684.66137.89, PMID: 15043342

[25] Sharma P Chatterjee P Mazumdar B . Recurrent carcinoma cervix presenting as metastatic splenomegaly: (18)F-FDG PET/CT findings in a rare scenario. Indian J Nucl Med. (2014) 29:200–1. doi: 10.4103/0972-3919.136608, PMID: 25210299 PMC4157207

[26] Shankar ST Panse M Goel A . Solitary splenic metastasis in a case of treated cervical cancer: a case report. Int J Reprod Contracept Obstet Gynecol. (2020) 9:2211–4. doi: 10.18203/2320-1770.ijrcog20201840

[27] Taga S Sawada M Nagai A Yamamoto D Hayase R . Splenic metastasis of squamous cell carcinoma of the uterine cervix: a case report and review of the literature. Case Rep Obstet Gynecol. (2014) 2014:798948. doi: 10.1155/2014/798948, PMID: 25152820 PMC4135132

[28] Theocharopoulos C Stanc G Douligeris CC Kontis EA Kopanakis N . Metachronous isolated splenic metastasis from cervical squamous cell carcinoma directly invading the stomach: A case report. Cureus. (2025) 17:e80304. doi: 10.7759/cureus.80304, PMID: 40201866 PMC11978419

[29] Valls C Serra J . Isolated splenic metastasis from uterine cervical adenocarcinoma. AJR Am J Roentgenol. (1992) 158:919–20. doi: 10.2214/ajr.158.4.1546619, PMID: 1546619

[30] Villalón-López JS Souto-del Bosque R Montañez-Lugo JI . Chávez-gonzález B.The rare occurrence of splenic metastasis of cervical cancer: A case report. Cancer+. (2020) 2:32–6. doi: 10.18063/cp.v2i4.358

[31] Gupta PK Lal P Tiwari A . A case report of carcinoma of uterine cervix throwing heterochronous metastasis to the skin, spleen, and pancreas: the role of multimodality treatment approach. J Egypt Natl Canc Inst. (2019) 31:8. doi: 10.1186/s43046-019-0009-9, PMID: 32372163 PMC13317717

[32] Vogel Y Schulte-Fischedick A Bauer H Zobel C Zienkiewicz T Pinsdorf T . Hämoglobinabfall nach einer Thrombolysetherapie bei einer 57-jährigen Patientin mit Schlaganfall und “erosiver Gastritis” [Hemoglobin drop after thrombolytic therapy in a 57-year-old stroke patient with “erosive gastritis”. Internist (Berl). (2020) 61:746–53. doi: 10.1007/s00108-020-00818-9, PMID: 32533196

[33] Gong H Zhao L Liu J . Protective effect of tretinoin on cervical cancer growth and proliferation through downregulation of pFAK2 expression. Environ Toxicol. (2024) 39:2732–40. doi: 10.1002/tox.24144, PMID: 38251951

[34] Han DJ Kim S Lee SY Moon Y Kang SJ Yoo J . et al. Evolutionary dependency of cancer mutations in gene pairs inferred by nonsynonymous-synonymous mutation ratios. Genome Med. (2024) 16:103. doi: 10.1186/s13073-024-01376-7, PMID: 39160568 PMC11331682

[35] Marcu ML Neacşu A Stoica C Bacalbaşa N Contolenco A Radu E . Clinical and pathological features of splenic metastasis from cervical squamous cell carcinoma. Rom J Morphol Embryol. (2017) 58:1157–64., PMID: 29556604

[36] Shu S Li Z Liu L Ying X Zhang Y Wang T . HPV16 E6-Activated OCT4 Promotes Cervical Cancer Progression by Suppressing p53 Expression via Co-Repressor NCOR1. Front Oncol. (2022) 12:900856. doi: 10.3389/fonc.2022.900856, PMID: 35875100 PMC9302044

[37] Liu J Li Z Lu T Pan J Li L Song Y . Genomic landscape, immune characteristics and prognostic mutation signature of cervical cancer in China. BMC Med Genomics. (2022) 15:231. doi: 10.1186/s12920-022-01376-9, PMID: 36333792 PMC9636686

[38] Wang SJ Yu XR Zhang QG Li YJ Fu CL Xu KL . Construction of a mouse model for myeloproliferative neoplasms and an evaluation system. Zhongguo Shi Yan Xue Ye Xue Za Zhi. (2023) 31:1113–8. doi: 10.19746/j.cnki.issn.1009-2137.2023.04.028, PMID: 37551485

[39] Brooks AN Choi PS de Waal L Sharifnia T Imielinski M Saksena G . A pan-cancer analysis of transcriptome changes associated with somatic mutations in U2AF1 reveals commonly altered splicing events. PloS One. (2014) 9:e87361. doi: 10.1371/journal.pone.0087361, PMID: 24498085 PMC3909098

[40] Fan Y Zhang L Sun Y Yang M Wang X Wu X . Expression profile and bioinformatics analysis of COMMD10 in BALB/C mice and human. Cancer Gene Ther. (2020) 27:216–25. doi: 10.1038/s41417-019-0087-9, PMID: 30787448

[41] Su L Liang W Lü Z Han X . PLXNA1 is highly expressed in hepatocellular carcinoma and affects patients’ survival and immune microenvironment. Nan Fang Yi Ke Da Xue Xue Bao. (2023) 43:1909–18. doi: 10.12122/j.issn.1673-4254.2023.11.11, PMID: 38081609 PMC10713469

[42] Chou B Prasad Venkatesulu B Coleman RL Harkenrider M Small W Jr . Management of stage I and II cervical cancer: a review. Int J Gynecol Cancer. (2022) 32:216–24. doi: 10.1136/ijgc-2021-002527, PMID: 35256406

[43] Lievens Y Guckenberger M Gomez D Hoyer M Iyengar P Kindts I . Defining oligometastatic disease from a radiation oncology perspective: An ESTRO-ASTRO consensus document. Radiother Oncol. (2020) 148:157–66. doi: 10.1016/j.radonc.2020.04.003, PMID: 32388150

[44] Bartl T Dorittke T Ciocsirescu C Knoth J Schmid M Grimm C . Oncologic outcome of metachronous oligometastatic recurrence in advanced cervical cancer patients after primary radio-chemotherapy. J Gynecol Oncol. (2025) 36(6):e99. doi: 10.3802/jgo.2025.36.e99, PMID: 40350705 PMC12636121

[45] Giannella L Di Giuseppe J Delli Carpini G Grelloni C Fichera M Sartini G . HPV-negative adenocarcinomas of the uterine cervix: from molecular characterization to clinical implications. Int J Mol Sci. (2022) 23:15022. doi: 10.3390/ijms232315022, PMID: 36499345 PMC9735497

[46] Ding Z Zhu H Mo L Li X Xu R Li T . FLT3L and granulocyte macrophage colony-stimulating factor enhance the anti-tumor and immune effects of an HPV16 E6/E7 vaccine. Aging (Albany NY). (2019) 11:11893–904. doi: 10.18632/aging.102494, PMID: 31881013 PMC6949056

[47] Xie Y Kong W Zhao X Zhang H Luo D Chen S . Immune checkpoint inhibitors in cervical cancer: Current status and research progress. Front Oncol. (2022) 12:984896. doi: 10.3389/fonc.2022.984896, PMID: 36387196 PMC9647018

[48] Li D Xu XX Yan DD Yuan SH Lou HM . Clinical significance of serum squamous cell carcinoma antigen in patients with early cervical squamous cell carcinoma. Zhonghua Zhong Liu Za Zhi. (2019) 41:357–62. doi: 10.3760/cma.j.issn.0253-3766.2019.05.007, PMID: 31137169

